# Which is more effective for the treatment of Acute Migraine Attack: Dexketoprofen, Ibuprofen or Metoclopramide?

**DOI:** 10.12669/pjms.342.13815

**Published:** 2018

**Authors:** Sinan Karacabey, Erkman Sanri, Sercan Yalcinli, Haldun Akoglu

**Affiliations:** 1 Sinan Karacabey, MD. Department of Emergency Medicine, Marmara University Pendik Training and Research Hospital, Pendik, Istanbul, Turkey; 2 Erkman Sanri, MD. Department of Emergency Medicine, Marmara University Pendik Training and Research Hospital, Pendik, Istanbul, Turkey; 3 Sercan Yalcinli, MD. Department of Emergency Medicine, Marmara University Pendik Training and Research Hospital, Pendik, Istanbul, Turkey; 4 Haldun Akoglu. Associate Professor, Department of Emergency Medicine, School of Medicine, Marmara University, Istanbul, Turkey

**Keywords:** Dexketoprofen, Ibuprofen, Metoclopramide, Migraine

## Abstract

**Objective:**

The aim of this study was head-to-head comparison of the efficacy and rate of adverse events of metoclopramide, ibuprofen and dexketoprofen for the acute treatment of migraine attack in the real-life conditions of a busy emergency department (ED).

**Methods:**

This was a prospective, observational, cross-sectional study. All patients who presented to the ED with a headache fulfilling the inclusion criteria were enrolled. All patients were treated by the attending emergency physicians in their daily routine. If an IV treatment in the ED was found indicated by the EP, they selected one of the options in the written departmental migraine treatment protocol.

**Results:**

During the study period, 54 patients met the inclusion criteria. The median change in the pain score was significantly different among treatment options (p<0.0001). The median pain score change at the end of the 30 minutes for treatment groups were 7.5 mm (IQR: 7.0-8.0), 5.0 mm (IQR: 4.75-7.0), and 7.0 mm (IQR: 6.0-7.25), respectively (p=0.0002). All three groups were found to be significantly different from each other in the post-hoc analysis.

**Conclusion:**

All drugs compared in this study are effective in the relief of migraine headache. However, IV dexketoprofen seems to be faster and more effective than metoclopramide and ibuprofen.

## INTRODUCTION

Headache is among the most common presentations to emergency department (ED), accounting for 1-4% of all visits.^1^ Migraine is a disabling neurological disorder which presents with headache of moderate to severe intensity. It is typically unilateral, pulsatile and generally associated with nausea and/or light and sound sensitivity and is often considered as one of the most severe type of acute pain, with up to 56.5% of those affected suffering moderate to severe disability.^2^ The prevalence of migraine is about 5-6% for males, and 14-17% for females.^1^ Diagnosis is based on clinical features, and diagnostic criteria proposed by the International Headache Society.^3^

Metoclopramide initially used to treat nausea in migraine attacks, however, it is now widely used as a single agent for pain relief in migraine attacks.^4^ Metoclopramide was compared to placebo in several studies^5^-^9^ showing that it is a reliable and effective treatment option. Recently two meta-analysis were published regarding the efficacy of metoclopramide.^4^,^10^ The value of the suggestion that metoclopramide may be used as the primary option in migraine headache has been disputed in the more recent meta-analysis due to lack of high quality clinical trials in the first meta-analysis.^4^,^10^ Ibuprofen is established as effective treatment option for acute migraine according to American Academy of Neurology (AAN) and American Headache Society (AHS) Guidelines^11^, and proved to be better than placebo.^12^,^13^ In a recent Cochrane review, NNT for 2-hour pain relief was 7.2 with 400 mg, and 9.7 with 200 mg ibuprofen.^12^ The strength of evidence for metoclopramide and ibuprofen for the treatment of acute migraine attack is high (Level B and Level A, respectively) according to AHS guidelines.^11^ Another and more recent treatment option, dexketoprofen, is a NSAID with rapid absorption rate and shorter time to maximum effect, and proposed as an effective treatment in acute migraine attacks.^14^ However, there are no trials comparing those three effective treatment options to date, to the best of our knowledge.

Studies regarding the treatment of headache and migraine, especially drug comparison trials, suffer from similar methodological problems. None of the pharmacological options available for acute migraine treatment are ideal for all patients.^15^ The clinical features of the patient and attack should be considered together in the decision-making process. Therefore, comparison of treatment options in real-life situations of a busy ED is needed and it will help to include the effect of physician gestalt and clinical features of the patients.

The aim of this study was head-to-head comparison of the efficacy and rate of adverse events of metoclopramide, ibuprofen and dexketoprofen for the acute treatment of migraine attack in the real-life conditions of a busy ED.

## METHODS

This prospective, observational, cross-sectional study was performed between the April 1^st^ and September 31^st^ of 2016, at the ED of a tertiary care hospital with an annual patient load of 54.000. Study protocol was examined and approved in by the Hospital Ethics Board (22.02.2016/26/05/01).

### Selection of Participants and Study Protocol

All patients who presented to the ED with a headache fulfilling the following inclusion criteria were enrolled and observed for study outcomes.

### Inclusion criteria

1)Non-pregnant
2)Age between 18 and 65 years
3)Have the diagnosis of migraine documented by a neurologist, or have migraine according to the IHS criteria (International Headache Society, Table-I)

**Table-I:** International Headache Society Diagnostic Criteria for migraine headaches without aura.

A.	At least five attacks fulfilling criteria B–D

B.	Headache attacks lasting 4-72 hours (untreated or unsuccessfully treated)


C.	Headache has at least two of the following four characteristics:

	1. Unilateral location
	2. Pulsating quality
	3. Moderate or severe pain intensity
	4. Aggravation by or causing avoidance of routine physical activity (e.g. walking or climbing stairs)

D.	During headache at least one of the following:

	1. nausea and/or vomiting
	2. photophobia and phonophobia

E	Not better accounted for by another ICHD-3 diagnosis.
4)Have an attack frequency from 2-6 episodes/month
5)No analgesic drug consumption within the last six hours before presenting to ED (NSAIDs, dopamine antagonists, serotonin receptor agonists, paracetamol etc.).

### Exclusion Criteria

Patients who:

1)Had concomitant headaches (e.g., tension-type, trigeminal autonomic cephalalgias, etc.)
2)Received rescue treatment in addition to routine migraine treatment in the ED
3)Refused to give consent.

All patients with headaches were evaluated, managed and treated by the attending emergency physicians (EPs) in their daily routine according to AAN guidelines. If an IV treatment in the ED was indicated by the EP, they selected one of the options in the written departmental migraine treatment protocol. This protocol included but not limited to the following three options: dexketoprofen 50 mg in 500 ml normal saline (NS) solution, ibuprofen 800 mg in 500 ml NS, and metoclopramide 10 mg in 500 ml NS. A researcher, who was blinded to the selected treatment option, documented patient’s migraine relief by a patient-based scoring system at three distinct time points where 0 was no pain and 10 was the imaginable worst pain.

These time points were

1)Beginning of the treatment (time zero)
2)15^th^ minute,
3)30^th^ minute.

The presence of any adverse events during the observation period was also documented by the researchers. Adverse events specifically sought for were akathisia, dystonia and pain at the injection site. Akathisia was defined as the feeling of restlessness and an urgent need to move. Dystonia was defined as involuntarily muscle contraction, causing repetitive or twisting movements. Pain at the injection site was defined as the presence of local pain at the infusion site.

### Outcome Measures

The primary outcomes of this study were the comparison of the change in the pain score (migraine relief) at the first, and second 15 minutes, and at the end of 30 minutes, among treatment options. The secondary outcome was the comparison of the rate of any adverse events.

### Statistical Analysis

Continuous variables were expressed with means, standard deviations (SDs) and 95% CIs, or medians and interquartile ranges (IQR), according to their distribution patterns. For the primary outcome, the magnitude of changes in VAS at different time points was compared with Kruskal Wallis test among treatment options. The post-hoc power of this test at the 30^th^ minutes was 0.95, and effect size (f) was 0.55. The power analysis was performed using G-Power for Mac OS X (V.3.1.9.2; Universitat Düsseldorf, Germany). A α-value of 0.05 was accepted as the nominal level of significance. All the statistical analyses were performed using MedCalc Statistical Software version 17.2 (MedCalc Software bvba, Ostend, Belgium; http://www.medcalc.org; 2017).

## RESULTS

**Fig.1:**
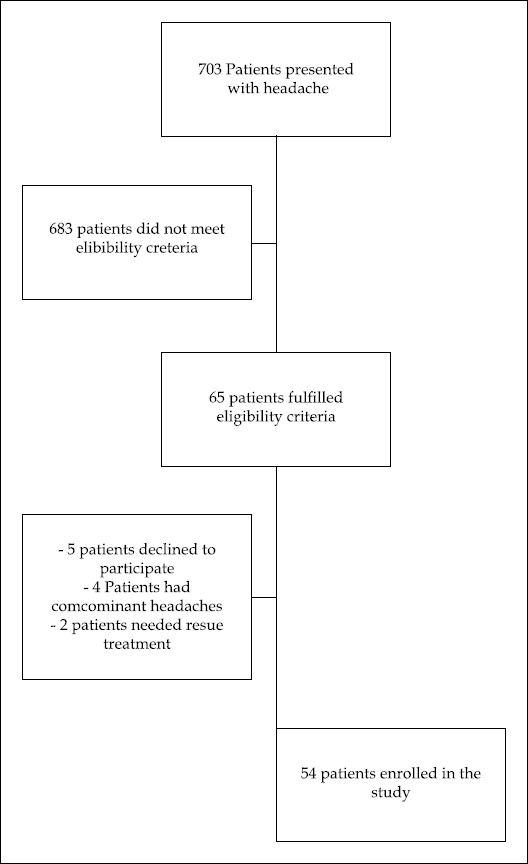
Flowchart of the study.

During the study period, 703 patients presented to the ED with headache, 638 (90.75%) did not meet the eligibility criteria, and 65 (9.25%) were enrolled to the study. Eleven (16.9%) patients were excluded from the study; therefore, the final study population was 54 patients (Fig.1). Mean age of the study subjects was 38.3 years (SD: 9.2; 95% CI: 35.8-40.9 years), and 83% (n=45) were women.

**Fig.2:**
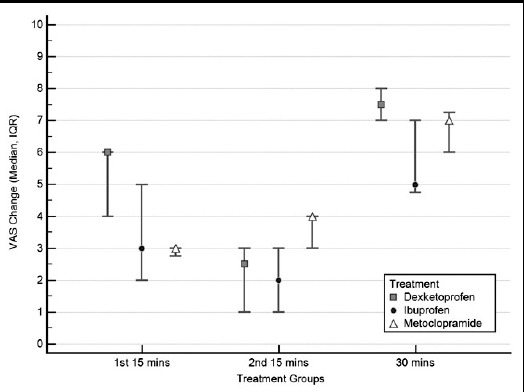
Comparison of the median VAS changes according to treatment groups.

The median changes in the pain score in the first 15 minutes for dexketoprofen, ibuprofen and metoclopramide groups were 6.0 cm (IQR: 4.0-6.0 cm), 3.0 cm (IQR: 2.0-5.0) and 3.0 cm (IQR: 2.75-3.00 cm), respectively. The median change in the pain score was significantly different among treatment options (p<0.0001, Table-II and Fig.2). Post-hoc analysis revealed that, median pain score change in the dexketoprofen group was significantly higher than other options in the first 15 minutes.

**Table-II:** Comparison of the VAS changes according to the treatment groups.

	ΔVAS (0-15min.)			ΔVAS (15-30 min.)			ΔVAS (0-30 min.)		
Treatment	Dex	Ibu	Met	Dex	Ibu	Met	Dex	Ibu	Met
N	20	17	17	20	17	17	20	17	17
Median	6.0	3.0	3.0	2.5	2.0	4.0	7.5	5.0	7.0
IQR	4.0-6.0	2.0-5.0	2.7-3.0	1.0-3.0	1.0-3.0	3.0-4.0	7.0-8.0	4.75-7.00	6.00-7.25
P*	<0.0001	0.0070	0.0002						

IQR: Interquartile Range, VAS: Visual Analog Scale, ΔVAS: VAS Difference, Dex: Dexketoprofen, Ibu: Ibuprofen; Met: Metoclopramide,

* Kruskal-Wallis Test.

The median pain score changes in the second 15 minutes for dexketoprofen, ibuprofen and metoclopramide groups were 2.5 mm (IQR: 1.0-3.0), 2.0 mm (IQR: 1.0-3.0), and 4.0 mm (IQR: 3.0-4.0), respectively (p=0.0070, Table-II). For this interval, median pain score change in the metoclopramide group was found to be significantly different than the other two options.

The median pain score change at the end of the 30 minutes for dexketoprofen, ibuprofen and metoclopramide groups were 7.5 mm (IQR: 7.0-8.0), 5.0 mm (IQR: 4.75-7.0), and 7.0 mm (IQR: 6.0-7.25), respectively (p=0.0002, Table-II). All three groups were found to be significantly different from each other in the post-hoc analysis. Therefore, the amount of the decrease in the pain score was in the following order from highest to lowest: dexketoprofen, metoclopramide, and ibuprofen. The adverse events are presented in Table-III. The rate of any adverse events were similar among groups (p=0.56, Table-III).

**Table-III:** Comparison of the presence of any adverse events according to treatment groups.

Adverse Events	Treatment N (%)		

	Dex (n=20)	Ib (n=17)	Met (n=17)
Akathisia	0 (0.0)	0 (0.0)	2 (100.0)
Dystonia	0 (0.0)	0 (0.0)	1 (100.0)
Injection site pain	3 (75.0)	1 (25.0)	0 (0.0)
Any adverse event	3 (15.0)	1 (5.9)	3 (17.7)

Dex: Dexketoprofen, Ibu: Ibuprofen; Met: Metoclopramide

## DISCUSSION

Our findings suggest that, all drugs compared in this study are effective in the relief of migraine headache; however, IV dexketoprofen seems to be faster and more effective than metoclopramide and ibuprofen. Dexketoprofen provided significantly more decrease in the VAS, both in the first 15 minutes, and at the end of 30 minutes. On the other hand, metoclopramide was significantly more effective in the second 15 minutes when compared to dexketoprofen and ibuprofen.

Metoclopramide is relatively inexpensive and commonly used for migraine attacks in the ED. In a randomized, double-blind, placebo controlled study comparing the efficacy of metoclopramide, prochlorperazine and saline (placebo) in acute migraine attack, metoclopramide was reported as an effective treatment.^7^ In this study, the median VAS was 8.5 mm at the beginning (IQR: 7.0-10.0 mm), and 5.6 mm at 60th minute (34% change) in the metoclopramide group. When compared to our study, median VAS of the patients at the beginning was similar (8.0 mm; IQR: 8.0-9.0 mm). We observed a similar decrease in pain score at the 15^th^ minute (5.0; IQR: 4.75-6.00), and greater decrease at the 30th minute (2.0; IQR: 1.0-2.0) with metoclopramide. Most of the clinical studies with metoclopramide were conducted by Friedman et al. They emphasized metoclopramide as the standard treatment of acute migraine attack in their studies, and used it as a comparative to the drugs in question.^16^-^19^ They reported a mean VAS decrease of 4.8^19^, 4.7^20^ and 4.7 mm^21^ at one hour for metoclopramide, which is similar to our findings. However, since we did not observe patients beyond 30^th^ minute, we don’t know if this effect extends to one hour in our study cohort, as well. We can conclude from our findings that metoclopramide is an effective option for the relief of migraine.

Ibuprofen is a non-steroidal anti-inflammatory drug (NSAID) which inhibits prostaglandin biosynthesis and has been used successfully mostly for the treatment of osteoarthritis and rheumatoid arthritis and also has given satisfying results in the treatment of muscle contraction headache and other muscular pains.^22^,^23^ Ibuprofen has been compared with paracetamol for migraine treatment and has been proved to be superior in reducing headache severity and duration, and obviating nausea and vomiting.^24^ However, our results showed that the pain score reduction achieved by ibuprofen was the least compared to other options for all time periods (first 15 minutes, second 15 minutes and 0-30 minutes). In a recent multicenter, double-blind, randomized, parallel-group, placebo-controlled, single-dose study comparing the fixed combination of acetaminophen, acetylsalicylic acid, and caffeine with ibuprofen for acute treatment of patients with severe migraine, both combination therapy and ibuprofen were found to be significantly more effective than placebo.^13^ However, combination therapy was reported as significantly faster and more effective than ibuprofen.^13^ In their latest upgrade of the Cochrane review, Rabbie et al. concluded that ibuprofen was an effective treatment for acute migraine which provides pain relief in about half of the patients, however, complete relief of pain and associated symptoms is low.^12^ Our findings also support those previous conclusions. We found that ibuprofen is significantly less effective for pain relief compared to dexketoprofen and metoclopramide.

There are two studies to date which investigated the effects of dexketoprofen in migraine headache. Gungor et al. conducted a prospective, randomized, double-blinded, placebo-controlled study of dexketoprofen in 224 patients to investigate its effectiveness in acute migraine headache.^14^ The base pain VAS of 112 patients was 80 mm (IQR: 70–90 mm), and the mean VAS decrease was 38.9 mm at 30 minutes with dexketoprofen (95% CI: 34.0-43.7). They concluded that dexketoprofen is an effective treatment with no serious adverse events compared to placebo.^14^ Turkcuer et al. also conducted a prospective, randomized, double-blinded study and compared the effectiveness of IV paracetamol with dexketoprofen in acute migraine attack in 200 ED patients.^25^ The base pain VAS was 89 mm (IQR: 77.5–95.0 mm), and the median VAS decrease was 55 mm at 30 minutes (IQR: 34–75 mm) in the dexketoprofen group.^25^ In our study the median pain score decrease in 30 minutes was 7.5 (IQR: 7.0-8.0) for dexketoprofen. Dexketoprofen was the most effective treatment both in the first 15 minutes and the entire 30 minutes, and was superior to ibuprofen and metoclopramide with no serious adverse events at 30 minutes. We think that the shorter time to pain relief with dexketoprofen may shorten the time to discharge of patients with migraine headache in the ED, which may be an advantage especially for crowded EDs.

### Limitations of the study

There are some limitations to this study. The selection criteria of IHS lead to most ED headache patients to be excluded from studies, and the same has occurred in this study as well. This may have limited the sample size of this study, therefore decreased the precision of our conclusions. Second, most studies of primary headache in the ED have found significant pain relief between 30-60 minutes. However, we were unable to evaluate this extended period in our study since most of our patients had relief of their pain and discharged from the ED. Third, the use and measurement of VAS would be a better estimate for the evaluation of pain, however, we preferred pain score for the ease of use. This may also have affected the precision of our conclusions. However, we found a significant difference between the efficacy of treatment options, and the calculated power to detect differences versus the alternative of equal means using an F test with a 0.05 significance level was 100%.

## CONCLUSION

Pain relief with IV dexketoprofen was significantly higher and faster than ibuprofen and metoclopramide for acute migraine headache without any significant increase in adverse events.

### Author`s Contribution

**SK** conceived, designed and manuscript writing.

**SK, SY** did data collection.

**ES** did statistical analysis.

**HA** editing of manuscript, did review and final approval of manuscript.
